# Maternal Serum C-Reactive Protein in Women with Preterm Prelabor Rupture of Membranes

**DOI:** 10.1371/journal.pone.0150217

**Published:** 2016-03-04

**Authors:** Martin Stepan, Teresa Cobo, Ivana Musilova, Helena Hornychova, Bo Jacobsson, Marian Kacerovsky

**Affiliations:** 1 Department of Obstetrics and Gynecology, Charles University in Prague, Faculty of Medicine Hradec Kralove, Hradec Kralove, Czech Republic; 2 Department of Obstetrics and Gynecology, Sahlgrenska academy, Sahlgrenska University Hospital, Gothenburg, Sweden; 3 BCNatal—Barcelona Center for Maternal-Fetal and Neonatal Medicine (Hospital Clinic and Hospital Sant Joan de Deu), Fetal i+D Fetal Medicine Research Center, IDIBAPS, University of Barcelona, Centre for Biomedical Research on Rare Diseases (CIBER-ER), Barcelona, Spain; 4 Fingerland´s Department of Pathology, Charles University in Prague, Faculty of Medicine Hradec Kralove, University Hospital Hradec Kralove, Hradec Kralove, Czech Republic; 5 Department of Genes and Environment, Division of Epidemiology, Norwegian Institute of Public Health, Oslo University, Oslo, Norway; 6 Biomedical Research Center, University Hospital Hradec Kralove, Hradec Kralove, Czech Republic; Xavier Bichat Medical School, INSERM-CNRS—Université Paris Diderot, FRANCE

## Abstract

**Objective:**

This study evaluated maternal C-reactive protein (CRP) as a predictor of microbial invasion of the amniotic cavity (MIAC) and histological chorioamnionitis (HCA) in women with preterm prelabor rupture of the membranes (PPROM) before and after 32 weeks of gestation.

**Methods:**

This study was a prospective observational cohort study of 386 women. Maternal serum CRP concentrations were evaluated, and amniotic fluid samples were obtained via transabdominal amniocentesis at the time of admission. Placentas underwent histopathological examination after delivery. MIAC was defined based on a positive PCR for *Ureaplasma* species, *Mycoplasma hominis* and *Chlamydia trachomatis* and/or positive 16S rRNA gene amplification. HCA was defined based on the Salafia classification.

**Results:**

Maternal CRP was significantly higher in women with MIAC and HCA (median 9.0 mg/l) than in women with HCA alone (median 6.9 mg/l), MIAC alone (median 7.4 mg/l) and without MIAC or HCA (median 4.5 mg/l) (*p*<0.0001). CRP was a weak predictor of the occurrence of MIAC and HCA before and after 32 weeks of gestation. Only the 95^th^ percentile of CRP and PPROM before 32 weeks exhibited a false-positive rate of 1%, a positive predictive value of 90% and a positive likelihood ratio of 13.2 to predict MIAC and HCA. However, the low sensitivity of 15% limits the clinical utility of this detection.

**Conclusion:**

CRP is a poor predictor of the occurrence of MIAC and HCA, even at early gestational ages.

## Introduction

Preterm delivery is a worldwide public health problem, and it occurs in approximately 6–12% of all pregnancies [1]. Preterm prelabor rupture of membranes (PPROM) is defined as amniotic fluid leakage before 37 weeks of gestation, and it represents approximately 30–40% of all preterm deliveries. PPROM is responsible for a substantial proportion of adverse neonatal complications associated with gestational age and risk of infection [2, 3].

Microbial invasion of the amniotic cavity (MIAC) is identified in 30–40% of PPROM, particularly at early gestational ages [4]. The presence of microorganisms activates an inflammatory response in the amniotic cavity, which is associated with early gestational age at delivery and a shorter latency to delivery [5].

Signs of histological chorioamnionitis (HCA) are also observed after placental examination in up to 60% of all preterm deliveries [6, 7]. HCA and funisitis are associated with neonatal composite morbidity, including chronic pulmonary disease [8] and adverse neurodevelopment outcomes [9, 10].

The active management of PPROM in the Czech Republic provides a short latency from membrane rupture to delivery in women with a PPROM diagnosis. This management scheme provides unique information on the subgroup of women presenting MIAC and HCA. We previously demonstrated that women with MIAC and HCA exhibited the greatest intra-amniotic inflammation [11, 12]. Similar results were observed in the fetal inflammatory response in the umbilical cord using interleukin (IL)-6 assessments[13]. Pregnancies complicated with MIAC and HCA exhibit the highest incidence of the subsequent development of early onset sepsis in newborns [14]. Notably, no differences were observed in the inflammatory responses in the amniotic cavity or fetal compartment in women with HCA or MIAC alone compared to a negative infectious group. Previous data suggest that the presence of MIAC and HCA represents the worse infectious scenario associated with the earliest gestational age at PPROM and the highest inflammatory response in amniotic fluid and umbilical cord. Therefore, the antenatal prediction of women with MIAC and HCA is crucial to improve the clinical management of women presenting with PPROM.

The only method to diagnose MIAC involves an invasive procedure, and placental information on HCA is not available antenatally. Therefore, non-invasive approaches were widely proposed (e.g., in maternal serum, cervico-vaginal fluid or ultrasound evaluations of the cervix and fetus [15–19]) to identify MIAC or HCA.

Evaluation of 27 biomarkers using a multiplex approach revealed differences in the concentration of IL-18, IL-1β and monocyte chemotactic protein-1 (MCP-1) in maternal serum between PPROM women with or without MIAC. Notably, these differences were only observed prior to 32 weeks of gestation [20]. However, this multiplex approach did not consider CRP in the analysis.

Maternal serum CRP has been proposed as a marker of infection and inflammation in several diseases [21–23] and MIAC and HCA. CRP exhibits a weak and contradictory association with these two conditions [19, 24, 25]. Howman et al. observed a significantly higher maternal inflammatory response when evaluating CRP in women with HCA [26]. However, a systematic review by Martinez et al. concluded that there was no evidence to support the use of CRP as a tool for the prediction of HCA [19]. No significant differences were reported when CRP levels were compared between PPROM women with or without MIAC [24]. To our knowledge, there are no previous studies evaluating the role of maternal CRP based on the presence of MIAC and HCA, HCA alone, MIAC alone and a negative infectious group.

It is clinically important to evaluate the role of CRP as a predictor of the worst infectious conditions, such as the occurrence of MIAC and HCA, because the information for maternal CRP associated with infection might induce changes in the management of PPROM in most clinical settings. Whether gestational age influences the predictive value of CRP for infectious conditions should also be investigated.

Therefore, the present study evaluated maternal CRP as a predictor of MIAC and HCA in women with PPROM before and after 32 weeks of gestation.

## Materials and Methods

This study was conducted as an observational prospective cohort study. The individuals enrolled in this study were pregnant women with PPROM between gestational ages 24+0 and 36+6 weeks who were admitted to the Department of Obstetrics and Gynecology, University Hospital Hradec Kralove, Czech Republic from January 2008 to December 2013. Gestational age was established based on the first trimester ultrasound scan.

Inclusion criteria were singleton pregnancies and maternal age ≥ 18 years. Women with signs of clinical chorioamnionitis, fetuses with an estimated weight <10^th^ percentile, the presence of either congenital or chromosomal fetal abnormalities, gestational or pre-gestational diabetes, gestational hypertension, preeclampsia and signs of fetal hypoxia were excluded from this study.

PPROM was diagnosed using a sterile speculum examination to verify the pooling of amniotic fluid and confirmed using a positive test for the presence of insulin-like growth factor-binding protein 1 (Actim Prom test, Medix Biochemica, Kaunianen, Finland) in vaginal fluid when necessary.

All women received prophylactic antibiotic treatment, and steroid therapy was administered when PPROM occurred from 24–34 weeks of gestation. Tocolysis was administered for 48 h during lung maturation.

The clinical guidelines of the Czech Republic recommends an active management of pregnancy when PPROM occurs later than 28 weeks of gestation. Labor is induced or Cesarean section is performed after lung maturation within 72 h following membrane rupture in all PPROM later than 28 weeks. The induction of labor or the performance of Cesarean section was indicated based on signs of fetal distress and/or severe vaginal bleeding and/or clinical chorioamnionitis or when high levels of maternal serum CRP were observed before 28 weeks. The remaining women were treated expectantly up to completed 28 weeks of gestation.

Written informed consent was obtained from all subjects. The University Hospital Hradec Kralove Institutional Review Board approved the collection and use of these samples and information for research purposes (March 19, 2008; No. 200804 SO1P).

Maternal blood and amniotic fluids were collected prior to treatment with antibiotics, steroids and tocolysis. Maternal blood was obtained via venipuncture of the cubital vein at admission. Maternal serum CRP was measured using a high-sensitivity immunoturbidimetric analysis (Modular PP analyzer, Roche, Basel, Switzerland), with a method sensitivity of 0.3 mg/L.

Amniotic fluid was obtained via ultrasound-guided transabdominal amniocentesis. Microbial analyses of amniotic fluid were based on specific polymerase chain reaction (PCR) for *Ureaplasma* species, *Mycoplasma hominis* and *Chlamydia trachomatis* and non-specific PCR for 16S rRNA. MIAC was determined based on a positive result of the PCR analysis for *Ureaplasma* species, *Mycoplasma hominis* and *Chlamydia trachomatis* and/or positive 16S rRNA gene amplification.

Placentas were collected after delivery and fixed in formalin, and tissue samples with placental membranes were embedded in paraffin. Tissue sections of placentas were stained using hematoxylin and eosin for standard histological examination. HCA was defined using the Salafia classification based on the presence of neutrophil infiltration in the chorion-decidua (Grades 3–4) and/or the chorionic plate (Grades 3–4) and/or the umbilical cord (Grades 1–4), and/or the amnion (Grades 1–4).[27]

### Detection of *Ureaplasma* species, *Mycoplasma hominis*, and *Chlamydia trachomatis*

DNA was isolated from amniotic fluid using a QIAamp DNA Mini Kit (QIAGEN, Hilden, Germany) according to the manufacturer’s instructions (using the protocol for the isolation of bacterial DNA from biological fluids). Real-time PCR was performed in a Rotor-Gene 6000 instrument (QIAGEN, Hilden, Germany) using the commercial kit AmpliSens^®^ C. trachomatis/Ureaplasma/M. hominis-FRT (Federal State Institution of Science, Central Research Institute of Epidemiology, Moscow, Russia) to detect the DNA from *Ureaplasma* species, *Mycoplasma hominis*, and *Chlamydia trachomatis* in a common PCR tube. We included a PCR run for the housekeeping gene beta-actin as a control to examine the presence of PCR inhibitors. The amount of *Ureaplasma* species DNA in copies/mL was determined using an absolute quantification technique that employs an external calibration curve. Plasmid DNA (pCR4, Invitrogen) was used to prepare the calibration curve [28, 29].

### Detection of other bacteria in the amniotic fluid

Bacterial DNA was identified using PCR targeting of the 16S rRNA gene with the following primers: 5`-CCAGACTCCTACGGGAGGCAG-3`(V3 region), 5`-ACATTTCACAACACGAGCTGACGA-3`(V6 region) [30, 31]. Each individual reaction contained 3 μL of target DNA, 500 nM of forward and reverse primers and Q5 High-Fidelity DNA polymerase (NEB, USA) in a total volume of 25 μL. The amplification was performed in a 2720 Thermal Cycler (Applied Biosystems, Foster City, CA, USA). The products were visualized on an agarose gel. Positive reactions yielded products of 950 bp, which were subsequently analyzed by sequencing. The 16S PCR products were cleaned and used in sequencing PCR reactions utilizing the above primers and the BigDye Terminator kit, version 3.1. The bacteria were typed using the sequences obtained in BLAST^®^ and SepsiTest^TM^ BLAST.

### Statistical analysis

All statistical analyses were performed using SPSS 21.0 for Mac OS X (SPSS Inc., Chicago, IL, USA) and GraphPad Prism 6.0 for Mac OS X (GraphPad Software, La Jolla, CA, USA).

Demographic and clinical characteristics were compared using non-parametric Kruskal-Wallis test, and data are presented as medians [interquartile range (IQR)]. Categorical variables were compared using the Chi-square test and presented as numbers [percentage (%)]. Spearman partial correlation was used to adjust the data for gestational age at admission or delivery. Spearman rank correlation test was used to determine the correlations between CRP concentrations and the microbial load of *Ureaplasma* species. Differences were considered statistically significant at *p* < 0.05, and *p*-values were obtained from two-sided tests.

## Results

### Clinical characteristics of the study population

**Fig 1:**
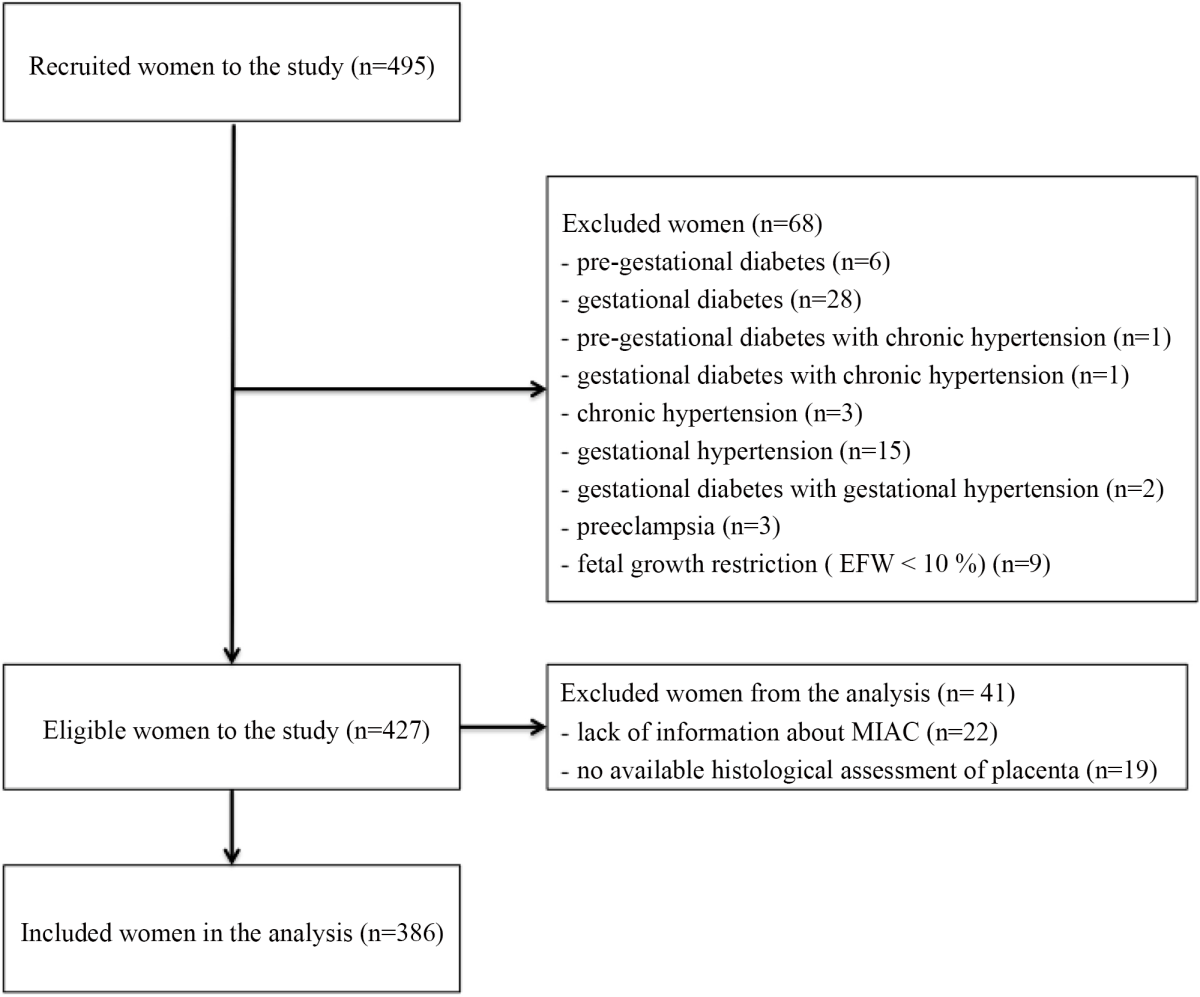
Inclusion of women with PPROM.

A total of 495 pregnant women with PPROM met the inclusion criteria and were included in the present study. Sixty-eight women were excluded because of pre-gestational diabetes, gestational diabetes, chronic hypertension, gestational hypertension, preeclampsia or fetal growth restriction in the fetus. Amniocentesis was not possible in 22 of the 427 women, and the results of histopathological assessments of the placenta were not available for 19 women. The remaining 386 women were included in the present study (Fig 1). Table 1 shows all microorganisms identified in the amniotic fluid.

**Table 1:** Microorganisms identified in the amniotic fluid.

Microorganisms identified in the amniotic fluid	Number of women
*Ureaplasma* species	78
*Mycoplasma hominis*	7
*Ureaplasma* species *+ Mycoplasma hominis*	9
*Ureaplasma* species *+ Chlamydia trachomatis*	5
*Streptococcus agalactiae*	4
*Haemophilus influenza*	3
*Staphylococcus hominis*	3
*Streptococcus α haemolyticus*	2
*Ureaplasma* species *+ Leptotrichia amnionii*	2
*Ureaplasma* species *+ Sneathia sanguinegens*	2
*Bifidobacterium* species	1
*Candida albicans*	1
*Enterococcus faecalis*	1
*Enterococcus faecim + Candida crusei*	1
*Enterococcus faecium*	1
*Fusobacterium nucleatum*	1
*Granulicatella elegans*	1
*Chlamydia trachomatis + Leptotrichia amnionii*	1
*Mycoplasma hominis + Lactobacillus* species	1
*Mycoplasma hominis + Peptococcus* species *+ Propionibacterium* species *+ Bacteroides* species	1
*Peptococcus* species *+ Propionibacterium* species	1
*Peptostreptococcus anaerobicus*	1
*Propionibacterium species + Peptostreptococcus* species	1
*Sneathia sanquinegens + Leptotrichia amnionii*	1
*Staphylococcus epidermidis*	1
*Staphylococcus haemofylus*	1
*Streptococcus anginosus*	1
*Streptococcus condemnatus*	1
*Streptococcus mitis*	1
*Streptococcus pneumoniae*	1
*Streptococcus* species	1
*Ureaplasma* species *+ Fusobacterium nucleatum*	1
*Ureaplasma* species *+ Lactobacillus crispatus*	1
*Ureaplasma* species *+ Lactobacillus species*	1
*Ureaplasma* species *+ Mycoplasma hominis + Fusobacterium nucleatum*	1
*Ureaplasma* species *+ Staphylococcus auricularis*	1
*Ureaplasma* species *+ Streptococcus oralis*	1
*Ureaplasma* species *+ Veillonela* species	1

The presence of MIAC and HCA was observed in 25% (97/386) of women. The presence of HCA or MIAC alone was observed in 36% (141/386) and 8% (32/386) of women, respectively. The remaining 29% (116/386) of women had neither MIAC nor HCA.

Table 2 presents maternal and neonatal characteristics according to the presence and absence of MIAC and/or HCA. The women with MIAC and HCA had the lowest gestational age and the lowest birth weight at admission and delivery. Latency from PPROM to delivery, gestational age at delivery and birth weight were not relevant clinical findings in this cohort of women because of the active management after 28 weeks of gestation [32].

**Table 2:** Maternal and neonatal characteristics in the different subgroups of PPROM pregnancies from 24+4 to 36+6 weeks of gestation.

	The presence of MIAC and HCA(n = 97)	The presence of HCA alone (n = 141)	The presence of MIAC alone (n = 32)	The absence of MIAC and HCA (n = 116)	*p-*value
Maternal age	31 (26–35)	32 (28–35)	31 (26–34)	30 (27–34)	0.13
Primiparous	33 (34%)	69 (49%)	17 (53%)	64 (55%)	**0.02**
Pre-pregnancy BMI	22.4 (20.1–25.1)	23.4 (21.1–26.3)	22.2 (19.6–24.2)	21.8(19.9–24.9)	**0.01**
Smoking	29 (30%)	21 (15%)	10 (31%)	15 (12%)	**0.002**
Gestational age at admission (week+days)	30+5 (27+6–33+6)	32+4 (31+1–35+0)	34+0 (33+0–35+6)	33+1 (31+5–35+1)	**<0.0001**
Gestational age at delivery (week+days)	31+1 (28+1–33+6)	32+6 (31+4–35+1)	34+1 (33+0–35+6)	33+2 (32+0–35+2)	**<0.0001**
Latency from PPROM to amniocentesis (h)	7 (4–12)	6 (4–11)	4 (3–6)	6 (3–10)	**0.002**
Latency from PPROM to delivery (h)	46 (24–83)	40 (22–76)	17 (9–51)	31 (14–63)	**0.001**
CRP levels at admission (mg/L)	9.0 (3.9–20.1)	6.4 (3.5–9.9)	4.3 (2.1–9.3)	4.5 (2–8.5)	**<0.0001**
WBC count ad admission (x10^9^/L)	13.1 (11.0–15.5)	11.7 (9.0–13.0)	11.7 (10.1–14.8)	12.0 (10.0–14.6)	0.46
Induction of labor	42 (43%)	37 (26%)	14 (44%)	29 (25%)	**0.006**
Vaginal delivery	69 (71%)	91 (65%)	26 (81%)	81 (70%)	0.29
Birth weight (grams)	1720 (1155–2125)	2080 (1640–2455)	2235 (1838–2518)	2280 (1748–2565)	**<0.0001**
5 min Apgar score < 7	10 (10%)	8 (6%)	0 (0%)	3 (3%)	0.06
10 min Apgar score < 7	6 (6%)	3 (2%)	0 (0%)	2 (2%)	0.13

Abbreviations: PPROM: preterm prelabor rupture of membranes. MIAC: microbial invasion of amniotic cavity. HCA: histological chorioamnionitis. BMI: body mass index. CRP: C-reactive protein. WBC: white blood cell. Continuous variables were compared using a non-parametric Kruskal Wallis test, and results are presented as medians (interquartile range). Categorical variables were compared using the Chi-square test and presented as numbers (%). Statistically significant differences are indicated in bold.

### Maternal serum CRP concentrations according to the presence or absence of MIAC and/or HCA

**Fig 2:**
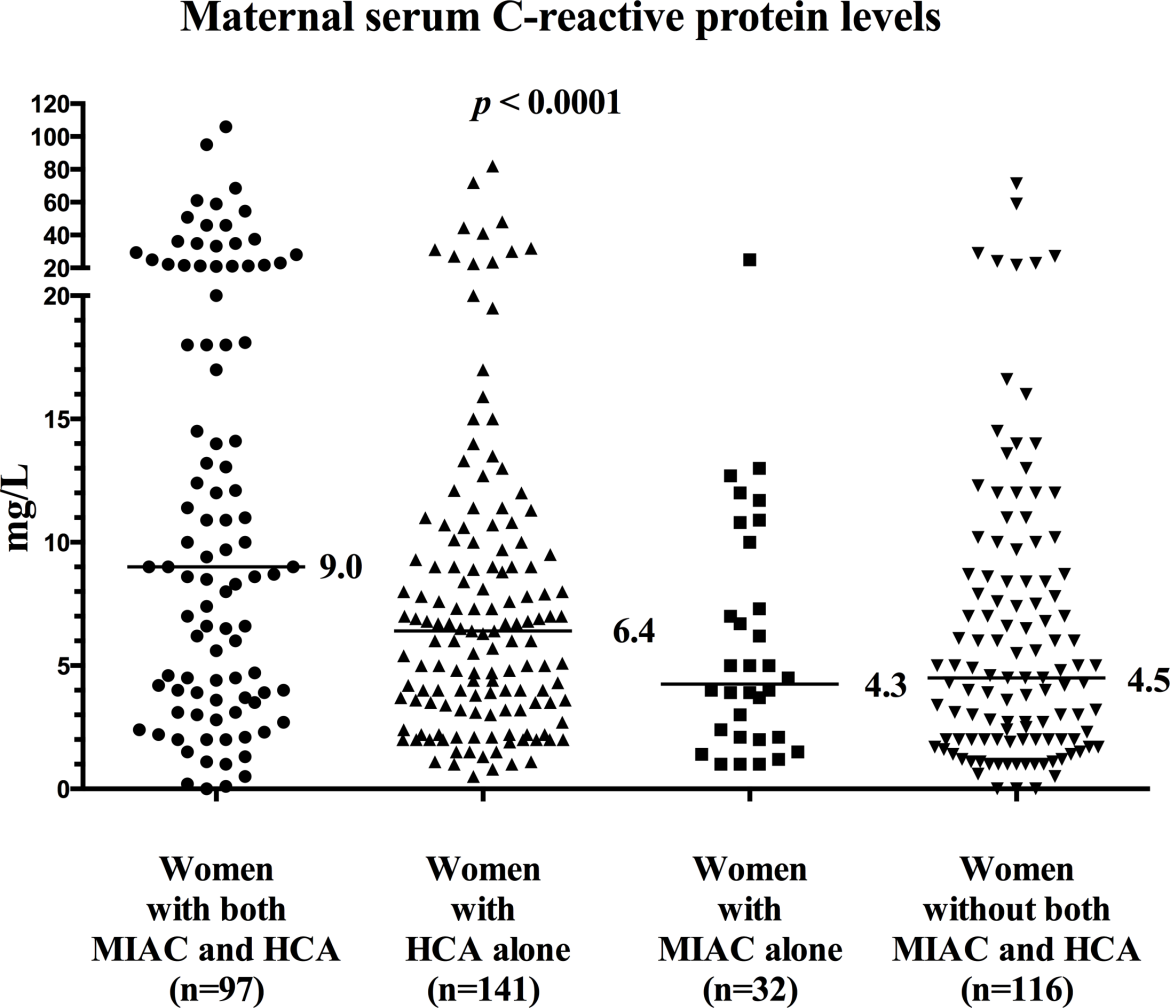
Maternal serum CRP concentrations (medians) according to subgroups of women with PPROM. Maternal serum C-reactive protein (CRP) concentrations according to the presence and absence of microbial invasion of the amniotic cavity (MIAC) and/or histological chorioamnionitis (HCA).

Women with MIAC and HCA exhibited the highest concentration of CRP [median: 9.0 mg/L (IQR: 3.9–20.1)] vs. women with HCA alone [median: 6.9 mg/L (IQR: 3.6–12.1)], MIAC alone [median: 7.4 mg/L (IQR: 3.6–15.6)] or with neither MIAC nor HCA [median: 4.5 mg/L (IQR: 2.0–8.6)]; (Fig 2) in crude analysis (*p* < 0.0001) and after adjustment for the gestational age of the sample (*p* < 0.0001).

Pregnancies with MIAC and HCA exhibited higher median CRP concentrations than women with HCA alone, MIAC alone, and neither MIAC nor HCA.

**Fig 3:**
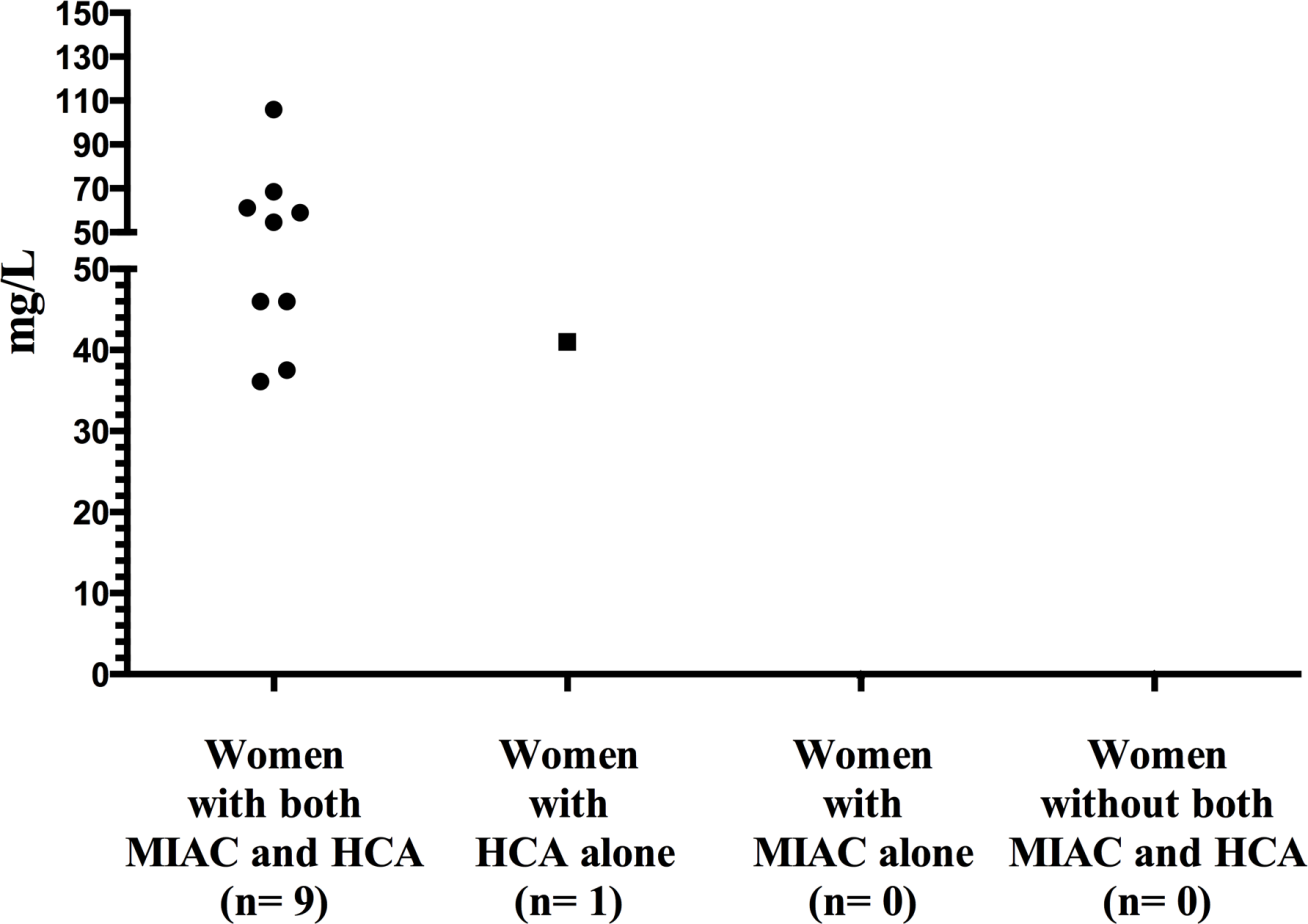
Maternal serum CRP concentrations (> 95^th^ percentile) based on the presence and absence of MIAC and/or HCA in PPROM < 32 weeks of gestation. Maternal serum C-reactive protein (CRP) concentrations (> 95 percentile) based on the presence and absence of microbial invasion of the amniotic cavity (MIAC) and/or histological chorioamnionitis (HCA) in women with PPROM below 32 weeks of gestation.

**Fig 4:**
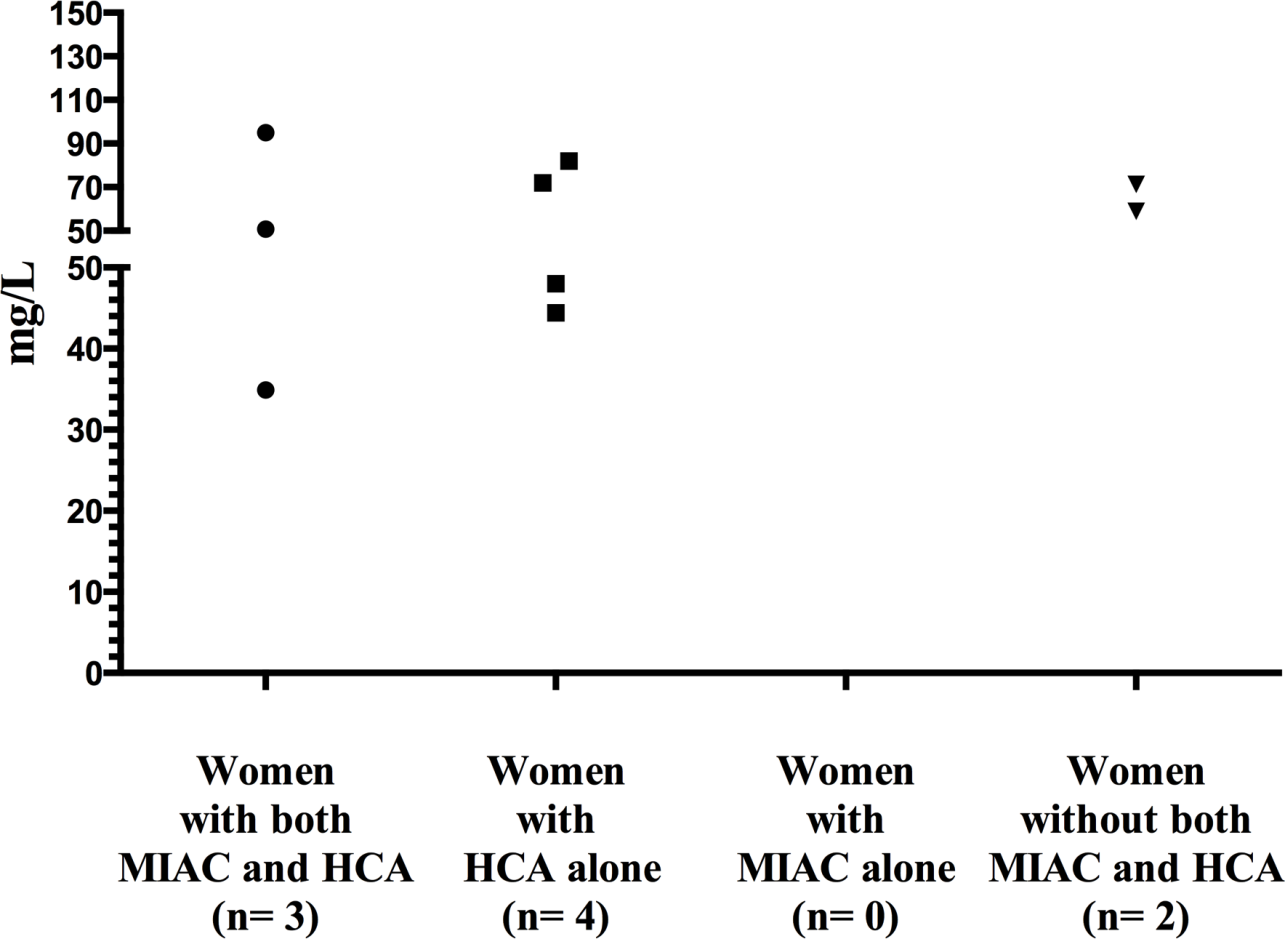
Maternal serum CRP concentrations (> 95^th^ percentile) based on the presence and absence of MIAC and/or HCA in PPROM > 32 weeks of gestation. Maternal serum C-reactive protein (CRP) concentrations (> 95 percentile) based on the presence and absence of microbial invasion of the amniotic cavity (MIAC) and/or histological chorioamnionitis (HCA) in women with PPROM above 32 weeks of gestation.

Table 3 shows the predictive value of CRP levels to identify women with MIAC and HCA below and above 32 weeks. Women with PPROM below 28 weeks were managed differently than women with gestational age longer than 28 weeks. Therefore, we analyzed these subgroups of women separately. The predictive values of CRP levels remained weak (Table 4). We examined the extreme values of CRP for the prediction of MIAC and HCA because the predictive values of CPR were poor (Table 5). Figs 3 and 4 shows the extreme values as represented by the 95^th^ percentile below and above 32 weeks of gestation. Supplemental information of the graphical distribution of CRP in the 90^th^ percentile below and above 32 weeks is provided in S1 Fig and S2 Fig. The small sample size in the subgroup of women with gestation age below 28 weeks prevented us from analyzing the extreme values of CRP.

**Table 3:** Prediction model for the presence of microbial invasion of the amniotic cavity and histological chorioamnionitis in PPROM below/above 32 weeks of gestation.

	Gestational age < 32+0 (n = 140)	Gestational age ≥ 32+0(n = 246)
Model	CRP > 6.45 mg/L	CRP > 6.95 mg/l
AUC	0.66	0.64
Sensitivity	63.1%	61.5%
Specificity	56.6%	62.6%
PPV	50.0%	24.0%
NPV	69.1%	90.5%
LR +	1.46	1.65

Abbreviations: AUC: Area under the curve. PPV: Positive predictive value. NPV: Negative predictive value. LR+: Positive Likelihood ratio.

**Table 4:** Prediction model for the presence of microbial invasion of the amniotic cavity and histological chorioamnionitis in PPROM based on the gestational age subgroups.

	Gestational age 24+0–28+0 (n = 50)	Gestational age 28+1–31+6 (n = 90)	Gestational age 32+0–36+6 (n = 246)
Model	CRP > 7.7 mg/L	CRP > 6.40 mg/L	CRP > 6.95 mg/l
AUC	0.70	0.62	0.64
Sensitivity	68.0%	60.0%	61.5%
Specificity	68.0%	52.4%	62.6%
PPV	68.0%	42.0%	24.0%
NPV	68.0%	68.9%	90.5%
LR +	2.12	1.25	1.65

Abbreviations: AUC: Area under the curve. PPV: Positive predictive value. NPV: Negative predictive value. +LR: Positive Likelihood ratio.

**Table 5:** Prediction model for the presence of microbial invasion of the amniotic cavity and histological chorioamnionitis in PPROM below/above 32 weeks of gestation using extreme CRP values.

	Gestational age < 32+0 (n = 18)	Gestational age < 32+0 (n = 10)	Gestational age ≥ 32+0 (n = 21)	Gestational age ≥ 32+0 (n = 9)
CRP cutoff levels	90^th^ percentile 21.6 mg/L	95^th^ percentile 34.7 mg/L	90^th^ percentile 21.6 mg/L	95^th^ percentile 34.7 mg/L
AUC	0.66	0.66	0.64	0.64
Sensitivity	22.8%	15.8%	17.5%	6.5%
Specificity	94.0%	98.8%	93.2%	97.0%
PPV	72.0%	90.0%	33.3%	33.3%
NPV	64.8%	63.1%	85.3%	82.3%
+ LR	3.8	13.1	2.6	2.2

Abbreviations: AUC: Area under the curve. PPV: Positive predictive value. NPV: Negative predictive value. +LR: Positive Likelihood ratio.

### Maternal serum CRP in women with genital mycoplasmas and other microorganisms in the amniotic fluid

No differences in maternal CRP concentrations were observed between women infected with genital mycoplasmas (*Ureaplasma species and/or Mycoplasma hominis*) [median: 6.6 mg/L (IQR: 3.5–12.1)] and women infected with other microorganisms [median: 7.1 mg/L (IQR: 2.63–14.2) *p* = 0.81].

### Maternal CRP concentrations and the bacterial load of *Ureaplasma* species

**Fig 5:**
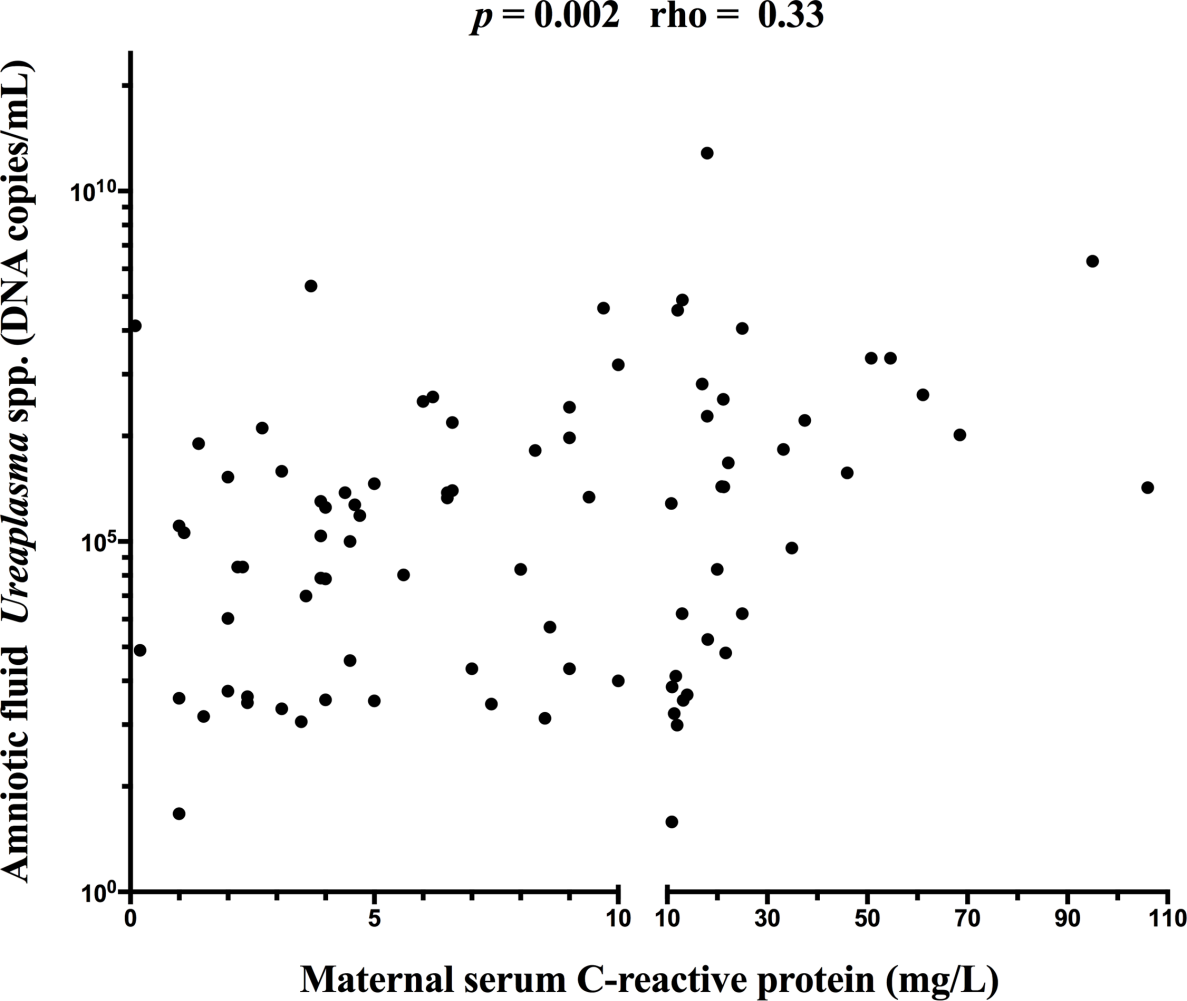
Correlation between microbial burden of *Ureaplasma* species in the amniotic fluid (copies DNA/mL) and maternal CRP concentrations.

A positive correlation between the microbial burden of *Ureaplasma* species in the amniotic fluid and maternal CRP concentrations was found (Spearman r = 0.33, *p* = 0.002; Fig 5).

### Microbial load of *Ureaplasma* species according to the presence of MIAC alone or MIAC and HCA

The amount of *Ureaplasma species* in amniotic fluid was determined in women who exhibited positive PCR for *Ureaplasma species* (70 women with MIAC and HCA and 16 women with MIAC alone). Women with MIAC and HCA (n = 70) had a higher microbial load of *Ureaplasma* species in amniotic fluid compared to women with MIAC alone (n = 16) [MIAC and HCA [median: 465000 (IQR): 3700–6667000)] vs. MIAC alone [median: 9333 (IQR: 597–235000), *p* < 0.005]].

## Discussion

The main finding of the present study was the weak association between maternal serum CRP and the occurrence of MIAC and HCA, even when different gestational ages were considered. Only CRP levels above the 95^th^ percentile in PPROM below 32 weeks of gestation accurately predicted the occurrence of MIAC and HCA. Unfortunately, this low sensitivity prevents the application of this finding in a clinical setting.

The worst scenario in PPROM is the presence of MIAC and HCA [4, 11, 33]. We demonstrated that the presence of MIAC and HCA represented the condition with the highest inflammatory response in the amniotic cavity and fetal compartment [12, 13]. However, the only method to diagnose MIAC is an invasive procedure, and histopathological information of HCA is not available antenatally. Therefore, the present study focused on the prediction of MIAC and HCA using a non-invasive approach, which involved the determination of a classical marker of infection, maternal serum CRP.

Previous studies reported the influence of CRP in pregnancies complicated with HCA[26] [34] [19], but few studies focused on the relationship with MIAC [24]. Howman et al. observed a significantly higher maternal inflammatory response when evaluating CRP in women with HCA[26]. The study population also included women with clinical chorioamnionitis. Therefore, the differences in CRP could partially reflect the inclusion of women with severe and late stage infections, when the maternal inflammatory response is primarily activated [35]. Contrary to Howman et al. and Laar et al., the inclusion of women with clinical chorioamnionitis did not exhibit differences when the role of CRP was evaluated to detect HCA [34]. Similarly, Martinez et al. conducted a review based on the evaluation of eight studies and did not observe data to support an influence of CRP on HCA [19]. A lack of association was also observed when evaluating maternal serum CRP between PPROM women with or without MIAC [24]. Nephelometry, turbidimetry with standard sensitivity[19, 34] and high sensitivity CRP nephelometry with lower limits of detection below 1.0 mg/L[26] were used to measure CRP. The variation in CRP results must be expected when different analytical techniques for CRP evaluation are used Nevertheless, it is unlikely that these variations affected the results of these studies [36].

Therefore, the data obtained in the present study revealed differences in CRP levels between different infectious subgroups of women with PPROM. CRP concentrations are gestational age-dependent[37], and differences in gestational age at sampling were observed between groups. Therefore, we adjusted the CRP concentrations for this confounding factor. CRP remained significantly higher in the subgroup of women with MIAC and HCA after adjusting by gestational age at sampling. However, the predictive value of CRP to identify the worst infectious scenario was weak, even at early gestational ages. We considered the extreme values represented by the 90^th^ and 95^th^ percentile to further explore the predictive value of CRP in a secondary analysis. The results demonstrated that a PPV of 90%, a FP rate of 1% and a +LR ratio of 13.1 predicted MIAC and HCA only when CRP levels were above the 95^th^ percentile and only PPROM below 32 weeks even when the extreme values of CRP were considered. However, the extremely low detection rate of 15% limits the clinical usefulness of this screening technique. These findings reveal that assessment of CRP levels was a poor predictor of MIAC and HCA, which highlights the importance of considering the amniocentesis as the only accurate method to identify this infectious condition. Therefore, these findings are interesting and clinically relevant.

Our previous study clearly demonstrated that the intraamniotic inflammatory response depended on the microbial load of *Ureaplasma* species. No differences were observed between the intensity of intraamniotic inflammatory response triggered by genital mycoplasmas and other microorganisms[38, 39]. The present study revealed an association between maternal CRP and the amount of *Ureaplasma* species in the amniotic fluid. We also did not observe differences in the intensity of maternal CRP between women with genital mycoplasma and other microorganisms. This finding is not consistent with Oh et al., who observed a higher inflammatory response in the amniotic cavity and maternal compartment in women with MIAC compared to the presence of genital mycoplasmas and other microorganisms[40]. These conflicting results may reflect the different methodologies used to identify bacteria in the amniotic fluid. Oh et al. used cultivation approaches in combination with the non-cultivation techniques (specific and non-specific PCRs) that were used in the present study.

One of the strengths of the present study is the active management of PPROM in our cohort, in which most women delivered within 72 h from membrane rupture. This management provides information on the placenta, which is consistent with the information obtained from amniotic fluid analyses. We also obtained microbial results from the amniotic fluid using modern non-cultivation technologies with a higher detection rate than classical cultivation approaches. We explored a large cohort of women in one tertiary center hospital with a clearly defined PPROM phenotype.

However, the present study also has some limitations. Maternal serum CRP analysis was performed only at admission, and we were unable to longitudinally analyze the progression of this protein until delivery. We also did not have information on IL-6 levels in amniotic fluid. Therefore, we could not evaluate the influence of maternal CRP on the intra-amniotic inflammatory response.

In conclusion, maternal CRP was a poor predictor for the identification of women with MIAC and HCA. Therefore, only the extreme cutoff levels of CRP, represented by the 95^th^ percentile, predicted the presence of MIAC and HCA below 32 weeks of gestation. However, the low sensitivity limits the clinical utility of this detection method. Therefore, there is no evidence to support the use of CRP as a tool for the prediction of the presence of MIAC and HCA, and the results suggest that amniocentesis is the most accurate tool for MIAC and HCA prediction in women with PPROM.

## Supporting Information

S1 Dataset (XLSX)

S1 Fig Maternal serum CRP concentrations (> 90^th^ percentile) based on the presence and absence of MIAC and/or HCA in PPROM < 32 weeks of gestation. Maternal serum C-reactive protein (CRP) concentrations (> 90 percentile) based on the presence and absence of microbial invasion of the amniotic cavity (MIAC) and/or histologic chorioamnionitis (HCA) in women with PPROM below 32 weeks of gestation.
(TIFF)

S2 Fig Maternal serum CRP concentrations (> 90^th^ percentile) based on the presence and absence of MIAC and/or HCA in PPROM > 32 weeks of gestation. Maternal serum C-reactive protein (CRP) concentrations (> 90 percentile) based on the presence and absence of microbial invasion of the amniotic cavity (MIAC) and/or histological chorioamnionitis (HCA) in women with PPROM above 32 weeks of gestation.
(TIFF)
