# Supplementary figures and images for: Maternal Serum C-Reactive Protein in Women with Preterm Prelabor Rupture of Membranes
Source: PLoS One. 2016 Mar 4;11(3):e0150217. doi: 10.1371/journal.pone.0150217 (PMC4778871; doi:10.1371/journal.pone.0150217)

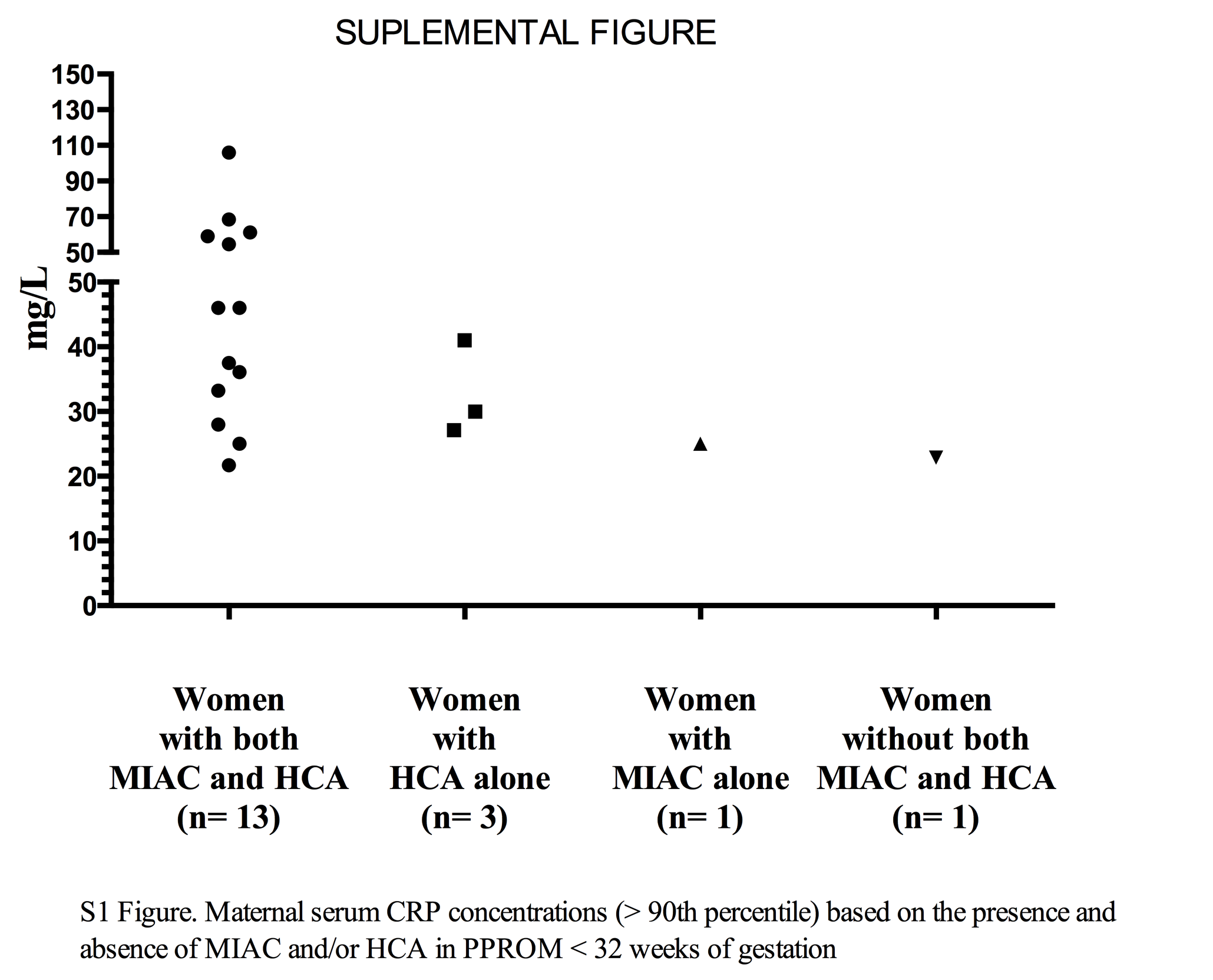

Supplement: S1 Fig — Maternal serum C-reactive protein (CRP) concentrations (> 90 percentile) based on the presence and absence of microbial invasion of the amniotic cavity (MIAC) and/or histologic chorioamnionitis (HCA) in women with PPROM below 32 weeks of gestation. (TIFF) [file pone.0150217.s002.tiff]

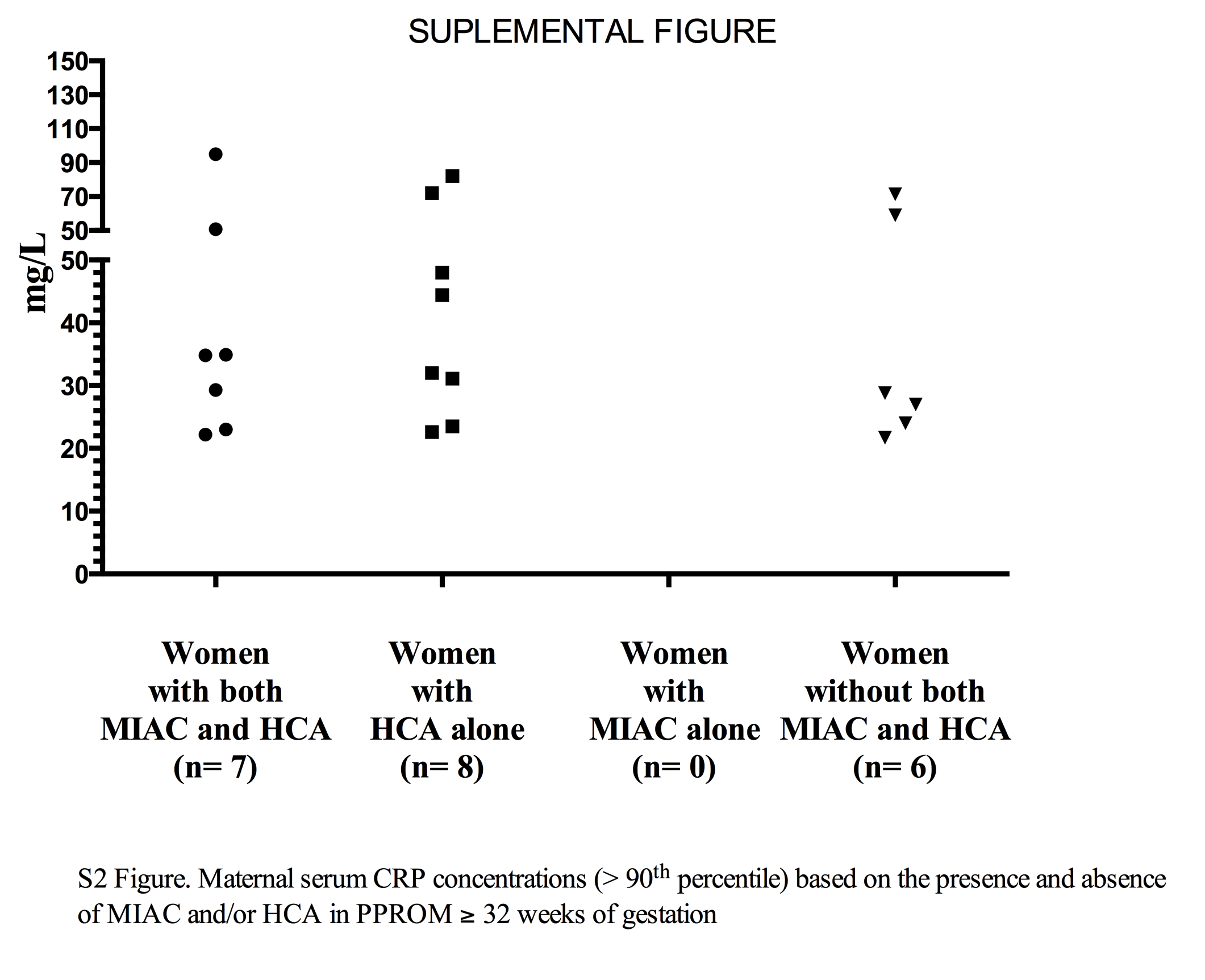

Supplement: S2 Fig — Maternal serum C-reactive protein (CRP) concentrations (> 90 percentile) based on the presence and absence of microbial invasion of the amniotic cavity (MIAC) and/or histological chorioamnionitis (HCA) in women with PPROM above 32 weeks of gestation. (TIFF) [file pone.0150217.s003.tiff]
